# Classification of wildfires in relation to land cover types and associated variables by applying cluster analysis: a case study in the Iberian Peninsula

**DOI:** 10.1007/s10661-025-14053-y

**Published:** 2025-05-03

**Authors:** Laura Serra, Pablo Juan, Carlos Díaz-Avalos, Pau Aragó, Somnath Chaudhuri, Sergio Trilles

**Affiliations:** 1 Research Group on Statistics, Econometrics and Health (GRECS), University of Girona, 17004 Girona, Spain; 2 IMAC, Department of Mathematics, Statistics Area, Universitat Jaume I, 12071 Castelló de La Plana, Spain; 3 Departamento de Probabilidad y Estadística, IIMAS, UNAM, Mexico City, México; 4 Servei Territorial de Medi Ambient. Conselleria de Medi Ambient d’infraestructures en Territori. Generalitat Valenciana, 46018 Valencia, Spain; 5 Center for Biomedical Research in Epidemiology and Public Health (CIBERESP), Madrid, Spain; 6 WorldPop Research Group, Department of Geography and Environmental Science, University of Southampton, Southampton, UK; 7 Institute of New Imaging Technologies (INIT), Universitat Jaume I, 12071 Castelló de La Plana, Spain

**Keywords:** Environmental covariates, Geographic clustering, Mixed-methods, Mclust, Wildfire patterns

## Abstract

Wildfires are a major environmental problem that have both economic and ecological impacts. Wildfires typically spread in a particular pattern, determined by factors such as the elements on the ground that catch fire or their geographic location. This study reports and discusses how wildfires in the Valencian Community, Spain, have been spatially grouped in recent years (from 2016 to 2020). It also characterizes each cluster in terms of location and land cover. An exploratory analysis of the environmental variables associated with wildfires has been conducted using finite Gaussian mixture models in R (R package mclust). The primary findings can be used to better understand the types of wildfires that occur in individual spatial zones. Some interesting cluster patterns in specific geographical areas, such as river basins, have also been reported. The method can identify clusters of fires by detecting areas with similar characteristics at the land use level. It also allows for the implementation of measures aimed at reducing the impacts of wildfires and can help in the extinction of wildfires based on the characteristics of all the fires grouped using spatial and land cover dimensions.

**Supplementary Information:**

The online version contains supplementary material available at 10.1007/s10661-025-14053-y.

## Introduction

Wildfires are one of the environmental hazards posing significant threats to wildlife and forests worldwide. According to Quílez Moraga (2019), the area covered by forests is growing, and the number of wildfires is decreasing. However, every year in the Valencian Community in Spain, a few wildfires account for most of the forest area burned. It is worth mentioning that even though the use of fire in agriculture is controlled and forest firefighters are quite efficient, in some cases, a few fires that started under adverse weather and land use conditions gave rise to significant wildfire incidences (de Rivera et al., 2020). Many scientific studies have analyzed the occurrence of wildfires in spatial domains aiming to understand the process and physical and biological factors that influence their incidence. For example, some studies have shown a strong relationship between the incidence of forest fires and climatic and meteorological variables in European countries (Hoinka et al., 2009; Moreno et al., 2014; Tedim et al., 2013; Venäläinen et al., 2014). A similar study by Aragó et al. (2016) investigated the incidence of forest fires in the province of Castelló, Spain, to identify risk factors associated with wildfires from 2001 to 2006.

Other studies (Carmo et al., 2011; Moreira et al., 2011; Nunes et al., 2016; Sebastián-López et al., 2007) have reported that human factors commonly linked to fire ignition are forest-agricultural or forest-urban interface constraints related to land use management. Thus, the characterization of wildfire events is necessary to model wildfire occurrence and to relate it to the spatial distribution of spatial varying factors, such as demographic indicators, land cover, and the presence of agricultural areas (Koutsias et al., 2010). Díaz-Avalos et al. (2016) explored and fitted statistical models using Bayesian statistics to identify relevant factors associated with the spatial variation of wildfire sizes using spatial marked point process methods.

On the other hand, recent research has shown that cluster analysis is a successful method for detecting regularities and can be used to design hybrid predictive models (Dong et al., 2016), as well as to study existential frequencies (Parente et al., 2016; Strauss et al., 2013). Many existing studies in the broader literature have examined the application of clusterization algorithms to explore and analyze the cause and spatial distribution of wildfire incidences. A research study (Castro et al., 2020) applied cluster analysis to review the spatial characterization of the distribution of wildfire events in mainland Portugal between 1996 and 2015. The research examined the breakdown of the causes of these fires over this period. A similar study reviewed the use of clustering techniques to assess wildfire impacts in storm activity conditions, especially lightning (Nikolay et al., 2016). Along the same line, Parente et al. (2016) proposed space–time clustering analysis techniques that correctly identify wildfire clusters in terms of their number and location despite eventual splitting of the datasets according to space and/or time.

Although some authors have conducted studies that examine spatial patterns and possible clusters in the domain of wildfire instances, this problem is still insufficiently explored. Moreover, most of the research published to date rarely analyzes the spatial distribution of wildfires concerning the type of land cover (Koutsias et al., 2010) and other meteorological parameters such as temperature and precipitation. Good knowledge of the causes of wildfires and their spatial–temporal distribution is crucial to produce reliable predictions of the number of fires and where and why they are expected to occur (Castro et al., 2020). This work aims to explore these little-studied aspects to understand the strategic measures required for preventing wildfires under the hypothesis that they are concentrated in specific areas according to land use. Accordingly, the patterns produced by wildfire incidents in the Valencian Community are analyzed with the aim of classifying wildfires based on associated covariables, such as land cover, causes, altitude, temperature, precipitation, and the area burned. The main objective is to test whether cluster analysis could be used as a tool to classify and separate wildfires based on the above mentioned covariables, to provide information useful to forest fire prevention authorities. Graphically, a specific accumulation of fires in different areas of the Valencian Community has been observed, with cluster analysis being the ideal tool to understand why those groups are formed. The methodological framework for analyzing wildfires in the Valencian Community consists of key stages: preparation (defining objectives and identifying the study area), data collection (wildfire incidents from 2016 to 2020 and environmental covariates such as land cover, causes, and meteorological data), and data processing (ensuring cleanliness and compatibility for analysis). Subsequently, cluster analysis is applied using finite Gaussian mixture models (GMM) through the R package mclust to identify and categorize wildfire patterns. Each cluster is then characterized based on its spatial distribution, land use type, and annual temporal variations. The analysis is validated with statistical tests to ensure the reliability of results. Finally, findings are interpreted within the context of wildfire management, with suggested future directions for research that include adapting the methodology for other regions and studying wildfire causes, including methods for detecting arsonists. The current study contributes to the relatively small amount of literature on cluster analysis methods to monitor wildfires in a broader picture. The proposed methodology is dynamic and can be effectively adapted and applied to other locations worldwide. The current analysis is based on finite GMM, implemented using the open-source R package mclust.

## Materials and methods

### Study site

**Fig. 1:**
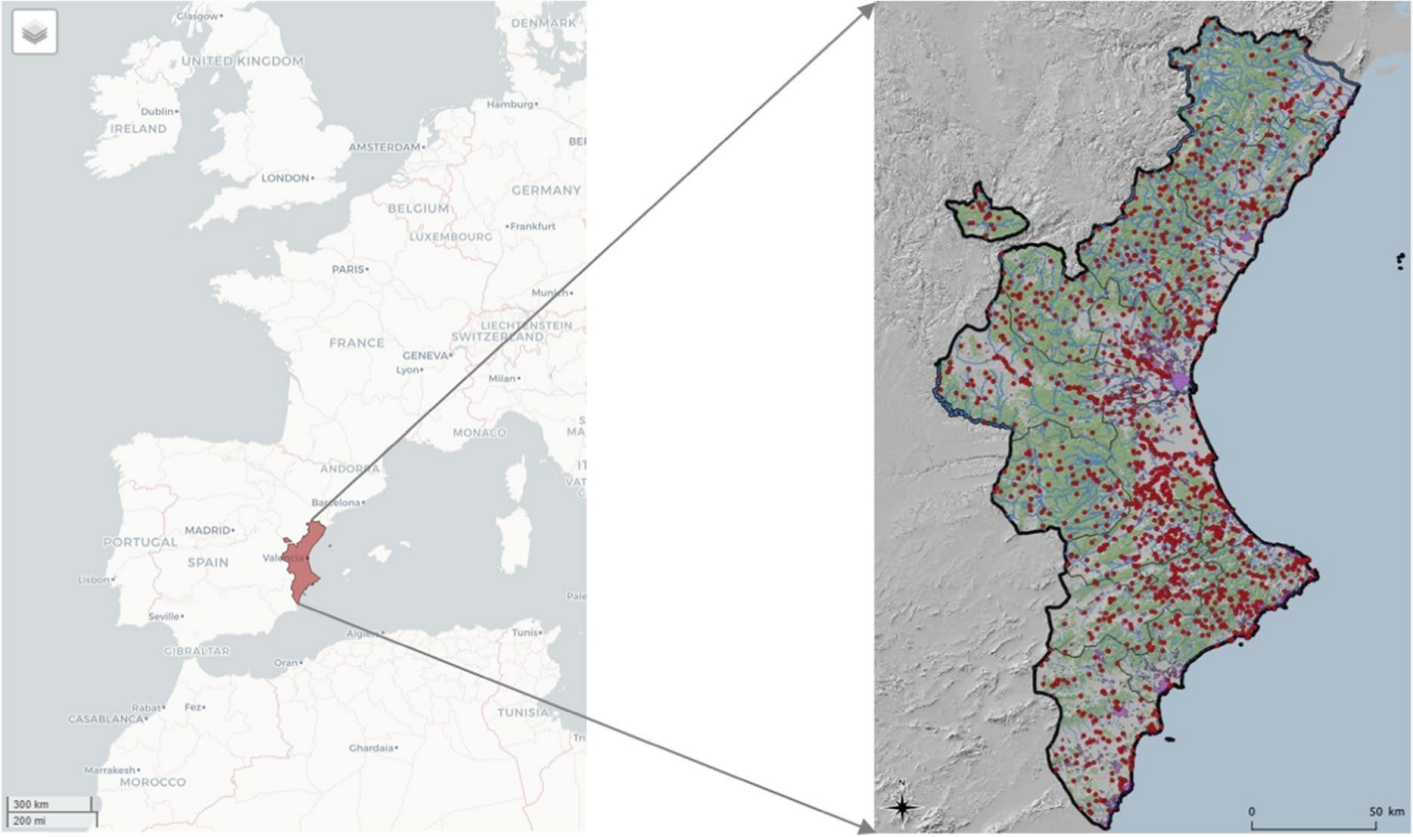
Geographical location of the study area—Valencian Community, Spain—and locations of wildfires in the study area from 2016 to 2020

The study area consisted of the Valencian Community, located on the eastern coast of the Iberian Peninsula. The region is bordered by Catalonia to the north, the Iberian Mountain System range to the west, and the Mediterranean Sea to the east. It is a region with a surface area of 2,324,500 ha, representing 4.6% of the Spanish national territory. The Valencian Community can be divided into a coastal zone, where fires have become a common tool for the management of agricultural biomass waste, and an inland zone, characterized by the presence of natural forest and rural areas, where the abandonment of farming areas is leading to the increase in forest land (Fig. 1).

### Data settings

**Fig. 2:**
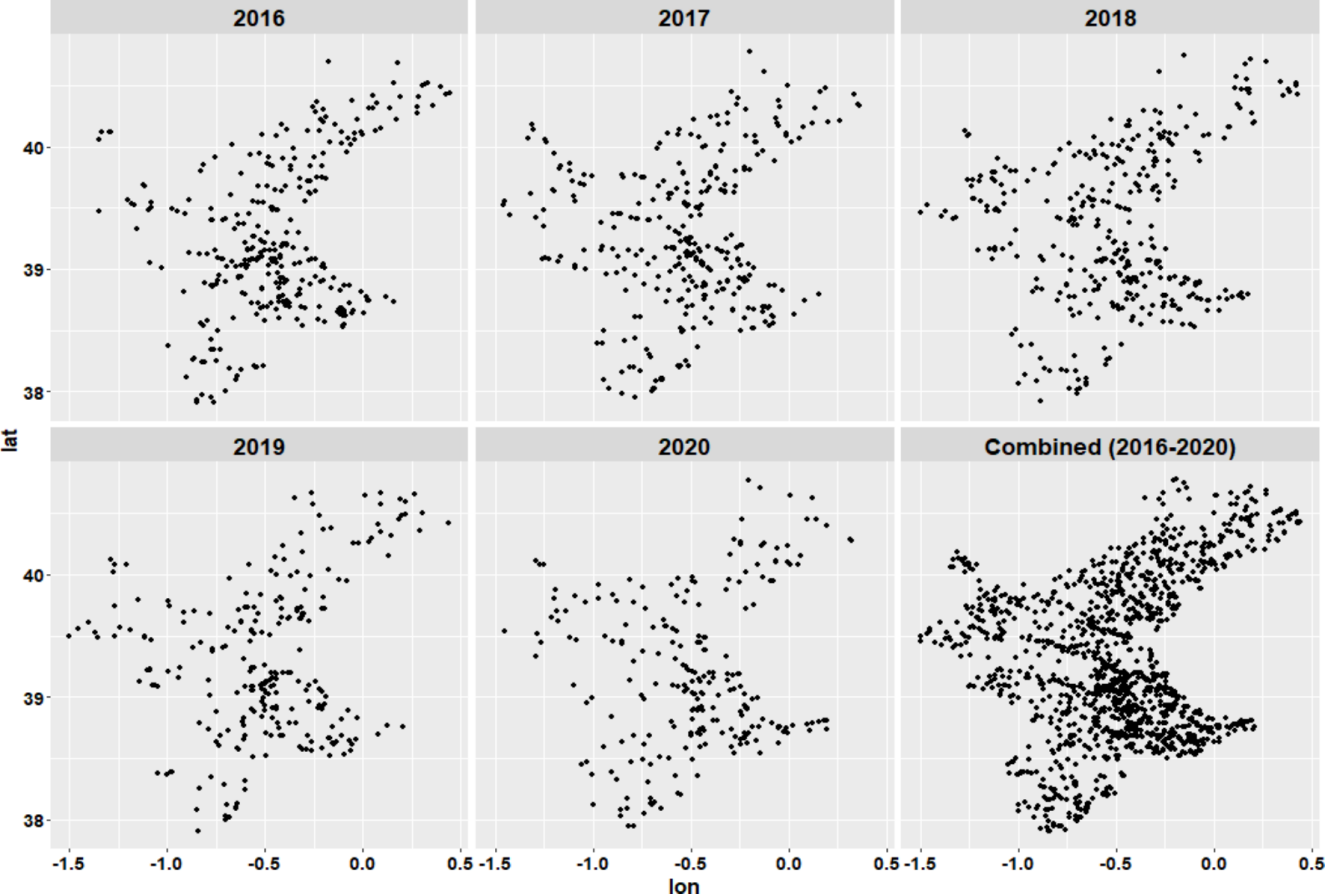
Annual spatial distribution of fires from 2016 to 2020, and all 5 years together throughout the Valencian Community

Data for this paper were obtained from the “Sistema integral de gestió d’incendis forestals” [Integrated wildfire management system] (Generalitat Valenciana, Spain). The data set includes the coordinates at which fire ignitions occurred, as well as information about the fire type, distinguishing between shrub and grassland fires, and wildfires. The elevation and meteorological variables values, such as annual average temperature and precipitation, were retrieved as raster files at a spatial resolution of 30 s using the R package *raster* (Hijmans & van Etten, 2012). These values were attached to the fire ignition data using GIS functions in R. The annual number of wildfire incidents in the study area was 341 in 2016, 346 in 2017, 375 in 2018, 272 in 2019, and 251 in 2020. The spatial distribution of the wildfires in the data set is shown in Fig. 2. The last plot represents a total of 1587 wildfires for the entire study period (2016–2020), which will be analyzed as a whole set as well as in individual years.

The globally interpolated climate data were obtained as raster images from NASA's Shuttle Radar Topography Mission (SRTM) database (Farr et al., 2007). Table 1 reports the summary statistics of the covariates for the complete data set. It provides information about four variables: (1) the burned area, distinguishing between shrub and grassland wildfires (not trees), wildfires (trees), and the total burned area in hectares; (2) wildfire altitude; (3) the annual temperature, differentiating between its mean value, the maximum, and the minimum, all in degrees Celsius; and (4) the annual precipitation in millimeters. The statistics shown in this table are the minimum and maximum values, the first and the third quartiles, the mean, the median, the variance, and the standard deviation of each covariate.

**Table 1:** Statistics of the covariates

	Burned area (tree)	Burned area (not tree)	Burned area (total)	Altitude	Temperature (mean)	Temperature (max)	Temperature (min)	Precipitation
Minimum	0	0	0	− 2	0	0	− 2	0
Maximum	1425.33	6146	6146	1385	18.3	32.8	6.9	671
Quartile 1	0	0	0.05	66.5	14.3	28.4	2.5	433
Quartile 3	0.2	1	3	621	17.3	30.4	5.9	501
Mean	6.18	18.63	23.30	357.24	15.42	28.75	4.04	454.82
Median	0	0	0.36	261	16.1	29.4	4.6	456
Stdev	55.52	201.23	210.77	324.72	2.92	4.38	2.11	90.42

### Methodology

#### Background of the method

GMM is a probabilistic generative model that assumes the data is a mixture of multiple Gaussian distributions of the form:

$$p\left(\text{x}|\lambda \right)=\sum_{i=1}^{M}{w}_{i}g(\text{x}|{\mu }_{i},\sum \nolimits_{i})$$

, each of which represents a latent cluster or group within the data. The mixture satisfies the restriction:

$$\sum_{i=1}^{M}{w}_{i}=1$$

The model estimates the parameters of the Gaussian distributions, such as mean and covariance, through maximum likelihood estimation or Bayesian inference (Reynolds, 2009). GMM is commonly used for cluster analysis and for other applications such as density estimation, anomaly detection, and dimensionality reduction. One of its advantages is its flexibility in modeling complex data distributions while offering a probabilistic interpretation of the results.

GMM can be used to analyze and predict the occurrence of wildfires. It can also be used to analyze the spatiotemporal patterns of wildfire occurrence, as well as the frequency, intensity, and size of wildfires (Chunyu et al., 2009; Han et al., 2017; Qian et al., 2018; Torabian et al., 2021; Yoon & Min, 2013; Zhao et al., 2011). By modeling the probability distribution of these patterns (Velizarova & Alexandrov, 2021; Zhang et al., 2017), GMM can be used to predict the likelihood of future wildfires in a particular region. Moreover, it can be used to identify the factors contributing to the occurrence of wildfires, such as weather conditions, topography, and human activities (Camarero et al., 2018; Shenoy et al., 2011; Wang et al., 2021; Ying et al., 2018). The insights from GMM analysis can be used to develop more effective wildfire prevention and management strategies.

The R-package mclust 5 (Fraley & Raftery, 2003; Scrucca et al., 2016) has been utilized in the current study, with the GMM approach being implemented. The results obtained allow to detect forest fire clusters based on input variables such as elevation, slope, and aspect. Little research has been conducted in which *mclust 5* has been used in forest fire prediction and analysis. Nonetheless, a study by Saxe et al. (2018) characterized and evaluated controls on post-fire streamflow response across western US watersheds. The mclust 5 package has been selected for application in the present study to predict the likelihood of fire ignition in specific areas based on the input variables, which facilitates proactive wildfire risk management and mitigation through cluster identification.

**Fig. 3:**
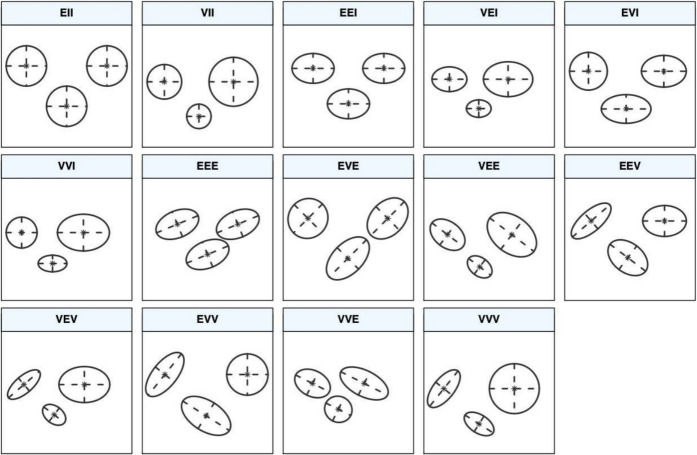
Ellipses of isodensity for each of the 14 Gaussian models obtained by eigen-decomposition in the case of three groups in two dimensions, *mclust*

In a model-based clustering approach, each component of a finite mixture density is usually associated with a group or a cluster. Most applications assume that all component densities arise from the same parametric distribution family, although this does not always have to be the case. For data ***x***_***i***_, …., ***x***_***n***_ in a D-dimensional space, a popular model is the GMM, a type of clustering algorithm that assumes a D-dimensional multivariate Gaussian distribution:

$$g\left(\text{x}|{\mu }_{i},\sum \nolimits_{i}\right)=\frac{1}{{(2\pi )}^\frac{D}{s}\sum \frac{1}{2}}\text{exp}\left\{-\frac{1}{2}{(\text{x}-{\mu }_{i})}{\prime}{\sum }^{-1}(\text{x}-{\mu }_{i})\right\}$$

for each component* i*, giving ellipsoidal clusters (Banfield & Raftery, 1993; Celeux & Govaert, 1995). It could be used for different possibilities, like the analysis included in Androniceanu et al. (2020). As its name implies, each cluster is modeled according to another Gaussian distribution. GMM is a probabilistic model that assumes all the data points are generated from a mixture of a finite number of Gaussian distributions with unknown parameters. These are used to represent normally distributed subpopulations within an overall population. The advantage of mixture models is that they do not require knowledge of which subpopulation a data point belongs to, but instead they allow the model to learn the subpopulations automatically (McLachlan et al., 2019). There are just two models in one dimension: *E* for equal variance and *V* for varying variance. The volume, shape, and orientation of the covariances in the multivariate setting can be constrained to be similar or variable across groups. Hence, 14 possible models with different geometric characteristics can be specified and are presented in Fig. 3. The dimensions considered in the analysis include elevation, slope, aspect, and combustible class. The use of GMMs to detect wildfires is a novelty from the last few years (Munshi, 2021) that can be further developed in future work with new methodologies. An integrated approach to finite mixture models is provided, with functions that combine model-based hierarchical clustering, the expectation–maximization (EM) algorithm for mixture estimation, and several tools for model selection. In particular, the EM algorithm is an affordable and numerically stable algorithm to implement, which has reliable global convergence under general conditions. However, the likelihood surface in mixture models tends to have multiple modes. Thus, initialization of EM is crucial because it usually produces sensible results based on reasonable starting values (Wu, 1983). This can be carried out using model-based hierarchical agglomerative clustering (MBHAC), which allows the underlying probabilistic model to be shared by both the initialization and model fitting steps. In addition, MBHAC is computationally efficient, as a single run provides the basis for initializing the EM algorithm for any number of mixture components and component-covariance parameterizations. There are different R packages to analyze clusters, for instance, *Mixtools* (Benaglia et al., 2009) or *Flexmix* (Leisch, 2004). The current analysis has been based on finite GMMs performed using the R package mclust (Fraley et al., 2014), which provides many functions to discover clustering. This software is a popular R package for model-based clustering, classification, and density estimation based on finite GMM.

#### Methodology followed

In this study, the *mclust* package was used to apply GMM for wildfire prediction and analysis as it provides a comprehensive strategy for clustering, density estimation, and discriminant analysis, which is the most important objective of the present study and has been applied in other studies on topics other than wildfires (Garcia-Rudolph et al., 2020; Zhang & Di, 2020). A variety of covariance structures obtained through eigenvalue decomposition are available. Functions for performing single E and M steps and for simulating data for each available model are also included. The functions included in the *mclust* package provide a data matrix from which the optimal number of components and the covariance parameterization are selected according to the Bayesian information criterion (BIC) for all the models and years considered (Scrucca et al., 2016). Hence, a good way to determine the final number of clusters is to consider the BIC value, which rises as the complexity of the model increases and decreases as the probability increases. So, the model with the lowest BIC is preferred. In the current study, the BIC value has been calculated for parameterized GMMs fitted by the EM algorithm initialized by model-based hierarchical clustering.

Additionally, within the *mclust* package, it is possible to visualize the cluster structure and the geometric characteristics (elliptic, spherical, etc.) induced by the estimated finite GMM. The data can also be projected onto a suitable dimension reduction subspace using the function *MclustDR()*, which implements the methodology introduced in Scrucca (2010).

Finally, *mclust* also provides GMMs with a simple interface for univariate and multivariate density estimation, which is very important as density estimation plays an important role in applied statistical data analysis and theoretical research. Finite mixture models provide a flexible semi-parametric model-based approach to density estimation, thereby making it possible to accurately approximate any given probability distribution using the *densityMclust()* function (Scrucca et al., 2016). Of the 14 potential models, as shown in Fig. 3, the standard models applied to analyze the data are as follows:

- VII,m: spherical and varying volume type of model with m clusters
- VEI,n: diagonal and equal shape type of model with n clusters
- VEE,p: ellipsoidal, equal shape and orientation type of model with p clusters
- VEV,q: ellipsoidal and equal shape type of model with q clusters
- EEV,r: ellipsoidal, equal volume and shape type of model with r clusters
- EVI,s: diagonal, equal volume and varying shape type of model with s clusters
- EII,t: spherical, equal volume type of model with t clusters
- EEI,x: diagonal, equal volume and shape type of model with x clusters

## Results

Cluster analysis groups spatially occurring events based on similarity or distance measures. GMM classify observations by likelihood, assuming clusters exist a priori. Table 2 shows the number of fires per cluster from 2016 to 2020. Figure 1 illustrates the geographical location of wildfire occurrences in the Valencian Community. In our analysis, clusters are formed in covariate space, meaning they do not necessarily exhibit a spatially clustered pattern. Cluster labels are arbitrary, and therefore, they are not consistent across years; for example, conditions for Cluster 1 in 2016 may differ from Cluster 1 in 2018. Nevertheless, our interest is on the characteristics of the clusters formed for each year, as they provide insight regarding the conditions for wildfire occurrence.

**Table 2:** Number of events by year

	Cluster					
Year	1	2	3	4	5	6
2016	48	45	51	104	55	34
2017	52	138	32	89	31	-
2018	167	38	69	40	61	-
2019	60	39	46	76	31	19
2020	52	76	22	42	58	-

**Fig. 4:**
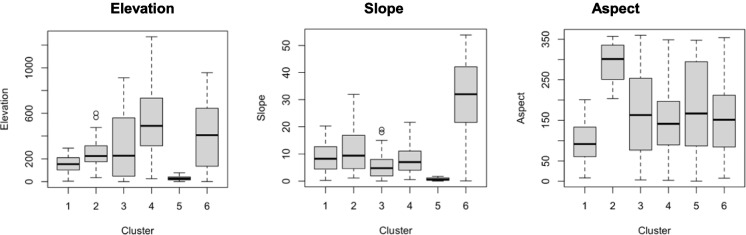
Elevation, slope, and aspect by cluster, for 2016

**Fig. 5:**
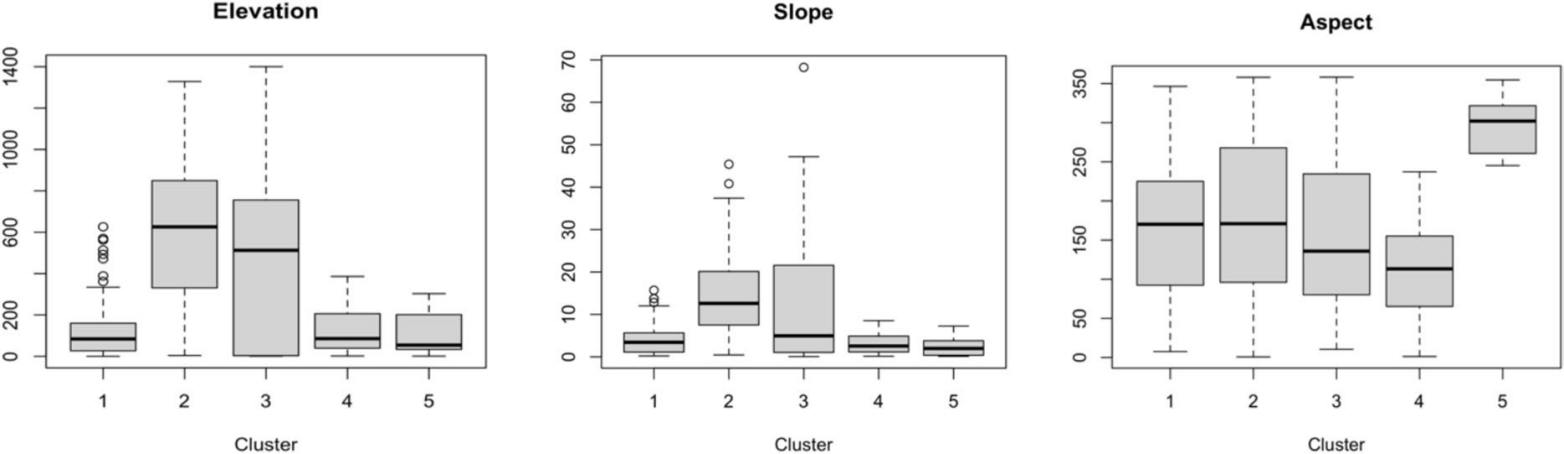
Clusters 1 to 3 (elevation, slope, and aspect) for 2017

**Fig. 6:**
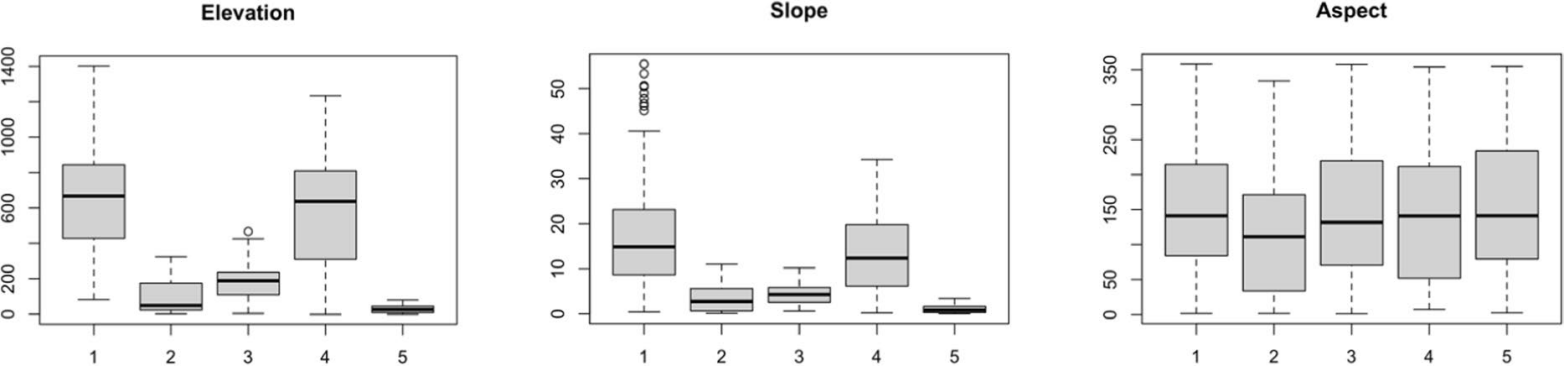
Clusters 4 to 6 (elevation, slope, and aspect) for 2018

**Fig. 7:**
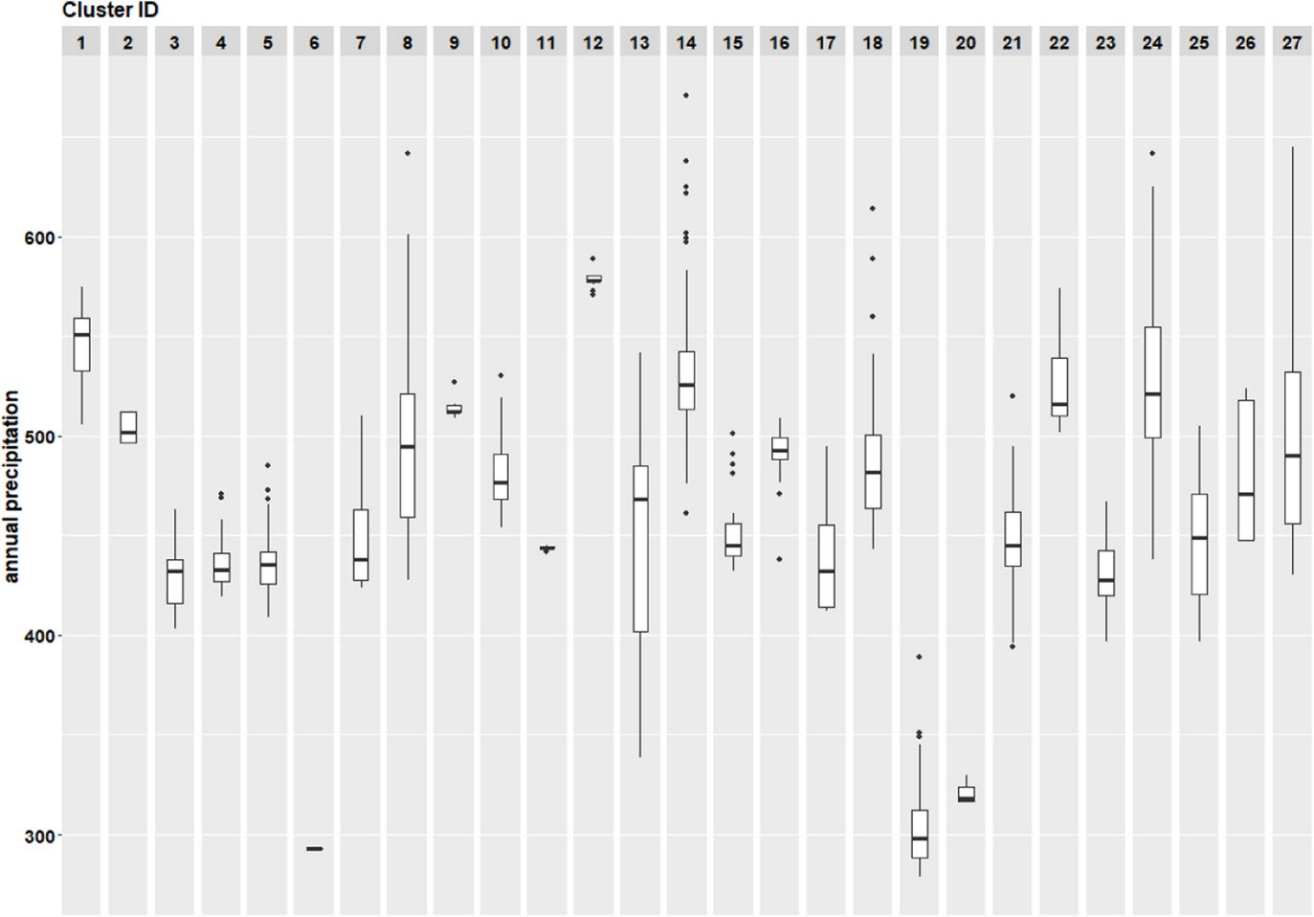
Boxplot of annual precipitation (2016–2020) per individual cluster. From clusters 1–6 (2016), from clusters 7–11 (2017), from clusters 12–16 (2018), from clusters 17–22 (2019), and from clusters 23–27 (2020)

**Fig. 8:**
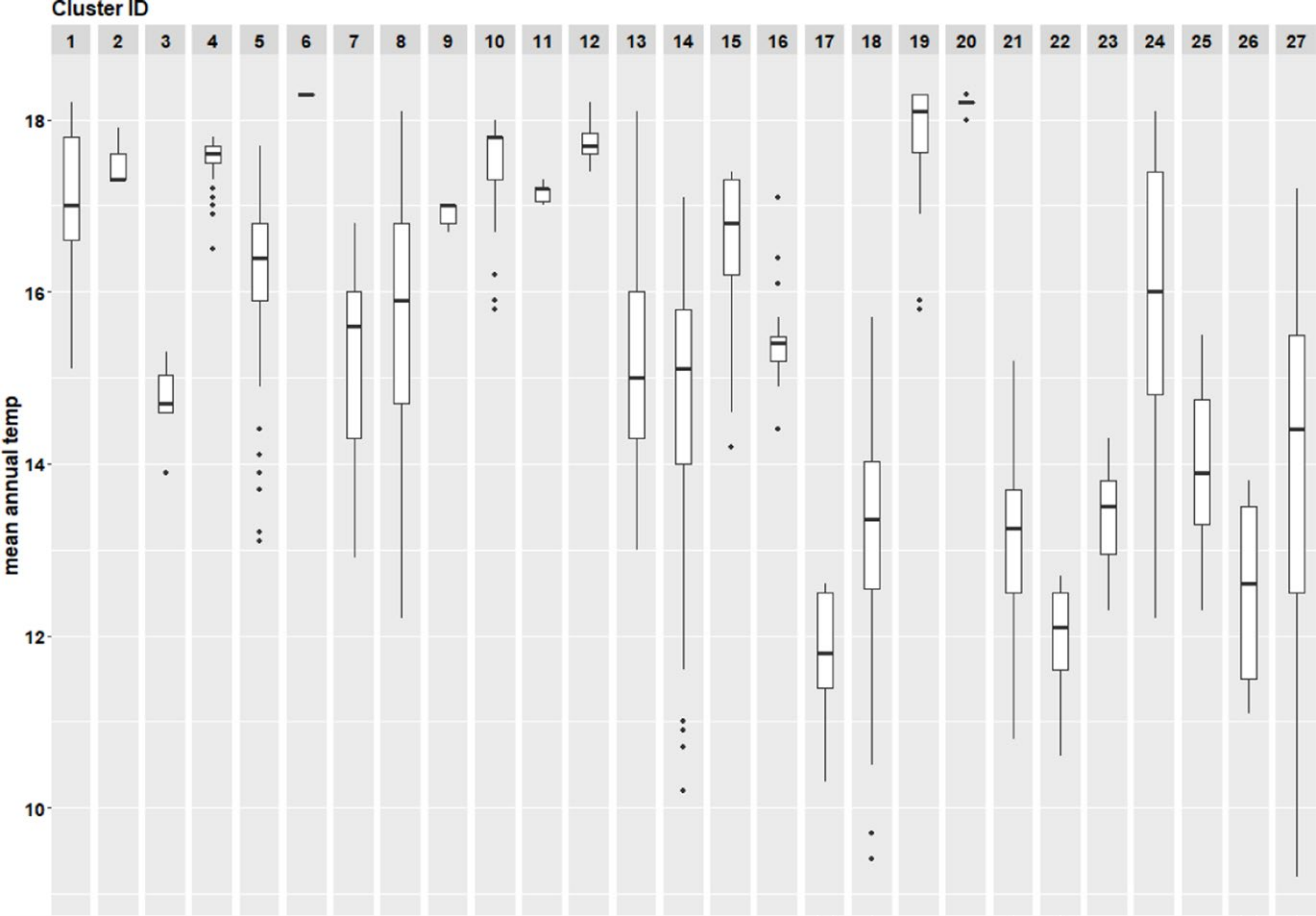
Boxplot of annual temperature (2016–2020) per individual cluster. From clusters 1–6 (2016), from clusters 7–11 (2017), from clusters 12–16 (2018), from clusters 17–22 (2019), and from clusters 23–27 (2020)

Figures 4, 5, 6, 7 and 8 present boxplots of cluster characteristics (elevation, slope, and aspect) for 2016, 2017, and 2018, respectively. Fires in Clusters 1, 2, and 5 occurred at low elevations and low to moderate slopes, with cluster 5 at the lowest elevations. Cluster 1 fires occurred on east-facing slopes, Cluster 2 on west-northwest-facing slopes, and Cluster 5 on slopes with a wider aspect range (east, southeast, south, and southwest). The wide range of aspect values for Cluster 5 suggests that those fires occurred on flat areas.

Most fires occurred in areas with Combustion Model Category 2, corresponding to grass and shrub stands, prevalent in Mediterranean regions during summer or drought periods. These areas are often found in agro-forestry systems or uncultivated fields.

For 2017, Clusters 1, 2, and 4 grouped most fires, primarily in Combustion Model Category 2 (grass height > 1 m). Cluster 2 also included fires in Categories 6 and 7. Clusters were geographically distinct, with Cluster 3 in the north and Cluster 4 in the south. Fires in Clusters 1 and 4 occurred at low elevations, low slopes, and east, southeast, west, and southwest-facing hills (Fig. 11).

For 2018, fires were classified into five clusters, with Clusters 1, 3, and 5 having the most memberships. Cluster 1 fires occurred in Combustion Model Categories 2, 6, 7, and 8 (grass and shrubs) at elevations of 500–800 m, mid-slopes, and south-facing hills. Clusters 3 and 5 corresponded to low-elevation, low-slope areas with east-to-south-facing hills.

For 2019, six clusters were identified, with most fires in Clusters 1 and 4. Fires in Clusters 4 and 5 ignited in Combustion Model Categories 6, 7, and 8, while others were primarily in Category 2 (Fig. 11).

For 2020, five clusters were identified, with most fires in Combustion Model Categories 2, 6, 7, and 8. Cluster 3 fires occurred in Categories 2, 3, and 5 (grass and slash). Fires in Clusters 1–4 occurred at elevations of 100–900 m, while Cluster 5 fires were at low elevations and nearly flat terrains (Fig. 11). For this year, there were no significant differences regarding aspect.

**Fig. 9:**
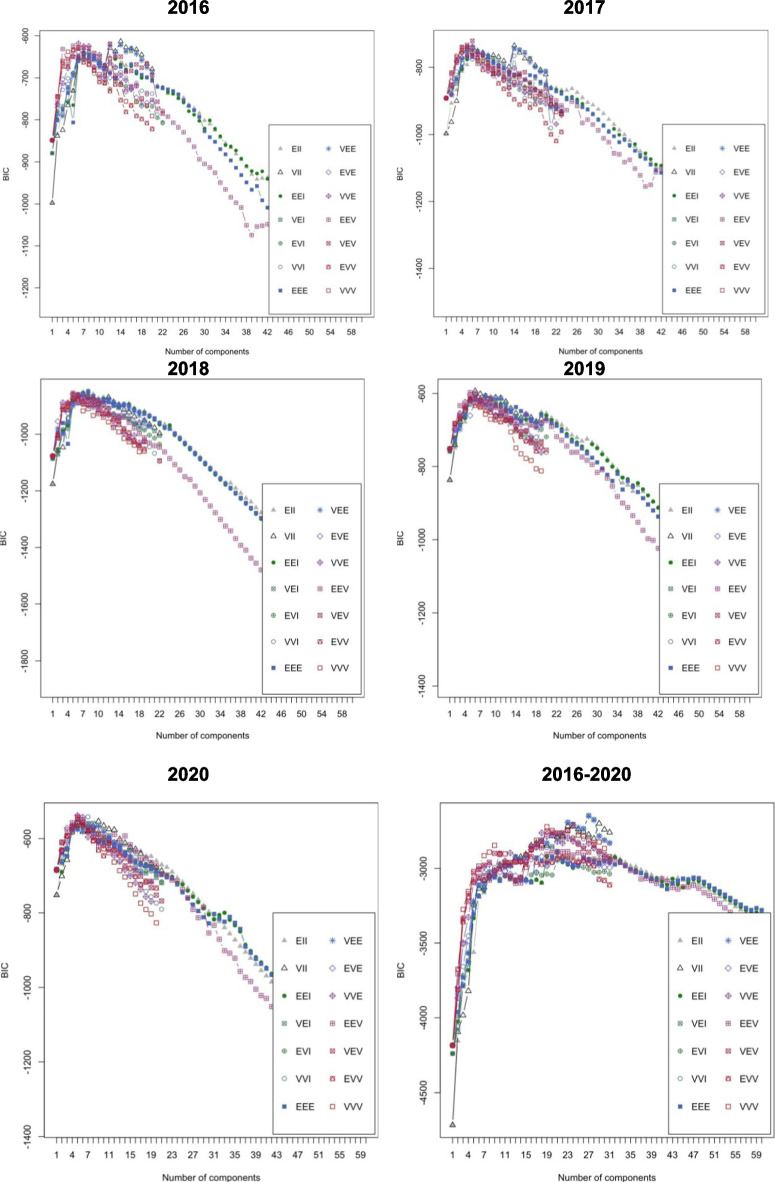
BIC values for the different models according to the model applied and the number of clusters considered from 2016 to 2020, and all 5 years together, using the function *densityMclust*. The different symbols represent events within each individual cluster detected

The spatial distribution can be observed by examining all wildfires that occurred during the study period (Fig. 1) and by analyzing the annual ignition points of these wildfires (Fig. 2). More specifically, looking at Fig. 2, although there are differences in the distribution of wildfires according to the year of occurrence, a certain grouping pattern is observed in specific parts of the study area. Some outbreaks with higher wildfire density are observed in the southern part of the Valencian Community, which has less forestry area but more borders between agriculture and forest. However, wildfires are primarily concentrated in the northern and central-western parts of the study area. This phenomenon could shed light on determining which factors related to the type of terrain or the cause of ignition are relevant for wildfires. The BIC influences the choice of cluster numbers and models by balancing model fit (how well the model explains the data) and model complexity (number of parameters). Lower BIC values indicate a better trade-off, guiding the selection of the optimal number of clusters and model type (e.g., VII, VVE, VEI). Variations in optimal models across years occur due to changes in data distribution, complexity, sample size, and noise levels. BIC values for the different models applied are shown in Fig. 9 for each year, according to the number of clusters considered. This figure shows that the best model is the one that reaches the maximum of the curve. In addition, Table 3 provides information about the BIC value, specifying the three models that perform the best every year and the optimum number of clusters, based on the BIC values (the minimum value being an absolute value). For example, simpler models like VII may suffice for years with less complex data, while more flexible models like VEI are preferred for years with intricate patterns. These variations reflect the dynamic nature of the data and its alignment with different model assumptions. Therefore, the VEI,12 model emerges as the best overall option for the period from 2016 to 2020, as it has the lowest cumulative BIC value (− 2671.84). This indicates that it provides the best balance of fit and complexity across the 5-year dataset, effectively capturing the underlying structure of the data while avoiding overfitting. While the optimal model varies by year depending on data characteristics, VEI,12 is the most robust choice for the entire period. The selected models differ according to the year, which means that the clusters and the shapes of these clusters in the Valencian Community during this period, 2016 to 2020, are different. This is the expected behavior due to the structure of wildfires and the burned zones. The best model and the optimal number of clusters for each year are reported in the first column of Table 3. The number of clusters in 2020 is lower than in all the previous years. This is because of the nationwide lockdown situation brought about by the ongoing global pandemic.

**Table 3:** Best BIC values from 2016 to 2020

Year	VII,14	VVE,6	VEI,12
2016	− 613.08	− 617.93	− 618.95
2017	VEV,6	VEV,5	VII,14
	− 720.71	− 733.24	− 734.49
2018	EII,8	EEE,8	EII,7
	− 846.57	− 848.09	− 851.45
2019	VII,6	EEV,5	VVE,6
	− 592.87	− 597.47	− 600.22
2020	VVE,5	VVI,7	VEV,5
	− 537.80	− 541.59	− 541.79
2016–2020	VEI,27	VEE,27	VEI,28
	− 2645.31	− 2651.23	− 2671.84

**Fig. 10:**
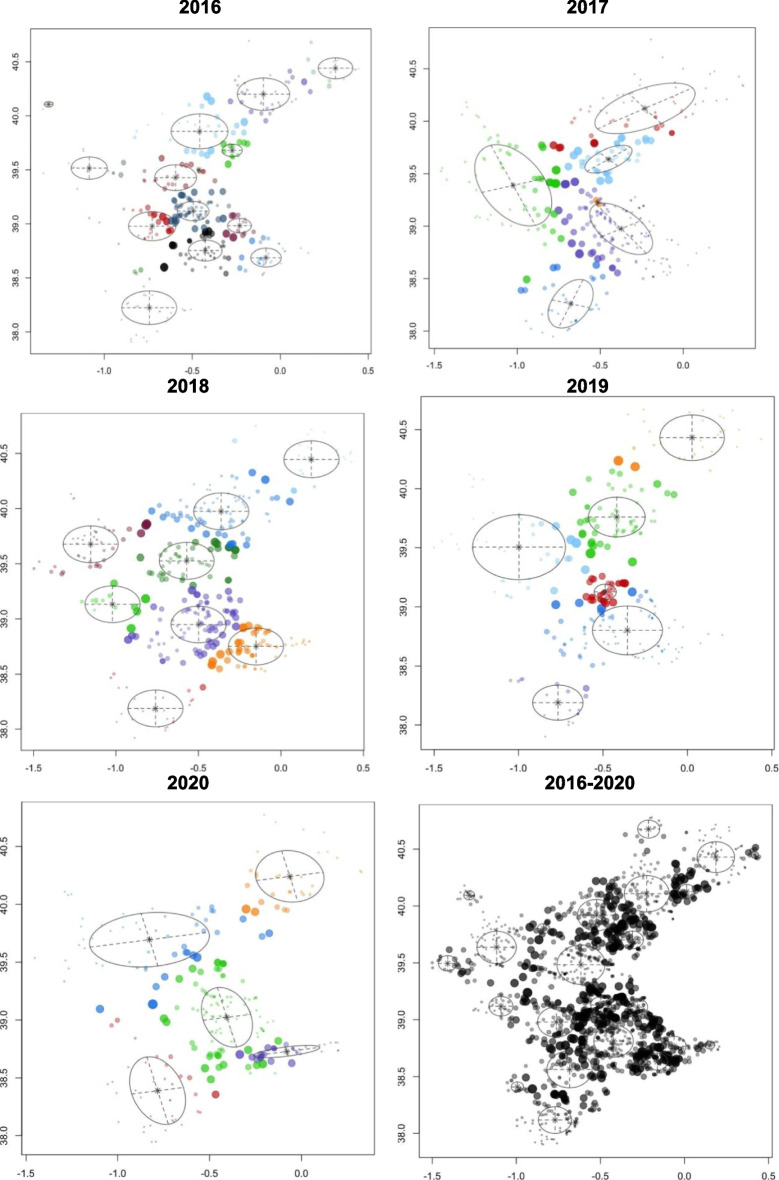
Cluster dispersion from 2016 to 2020, and all 5 years together, using the function *Mclust* over the surface area of the Valencian Community (different symbols represent events within individual clusters detected and each color represents unique clusters)

Figure 10 illustrates cluster density and dispersion, with circle size indicating ignition point dispersion. Cluster shapes and structures varied annually, reflecting diverse fire behaviors and characteristics.

**Fig. 11:**
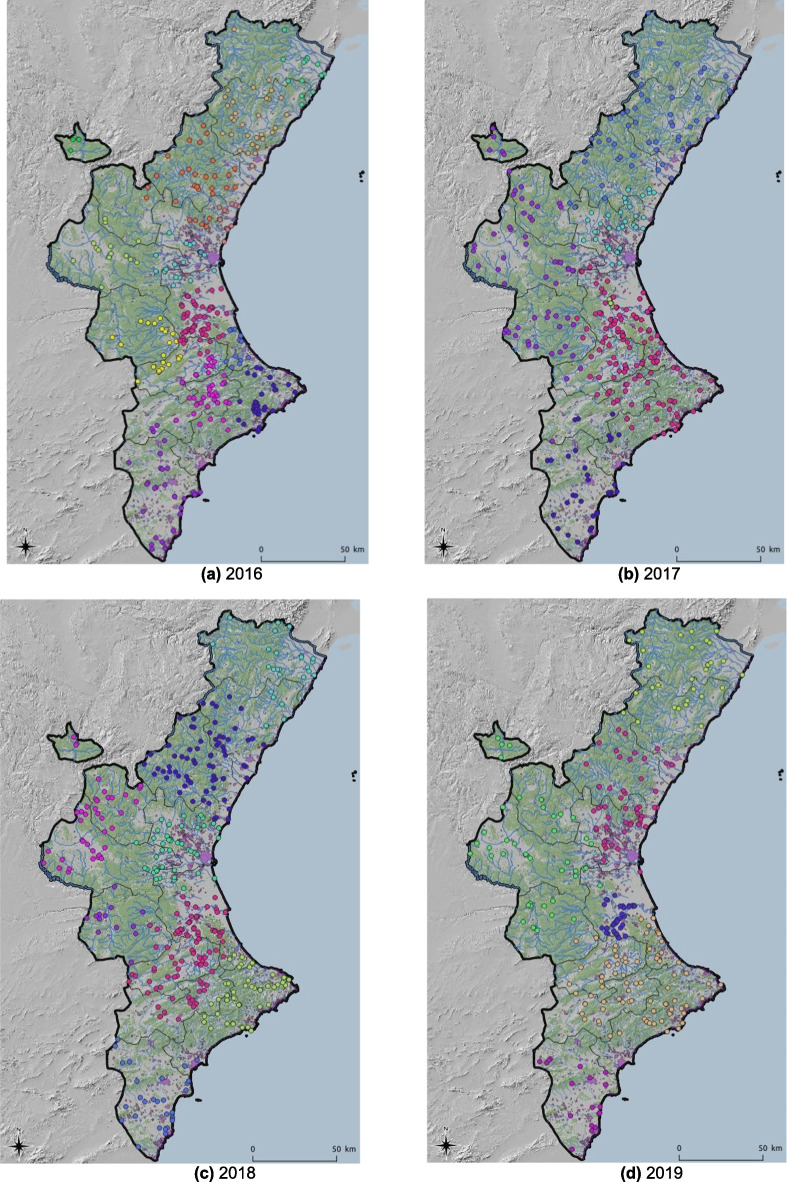

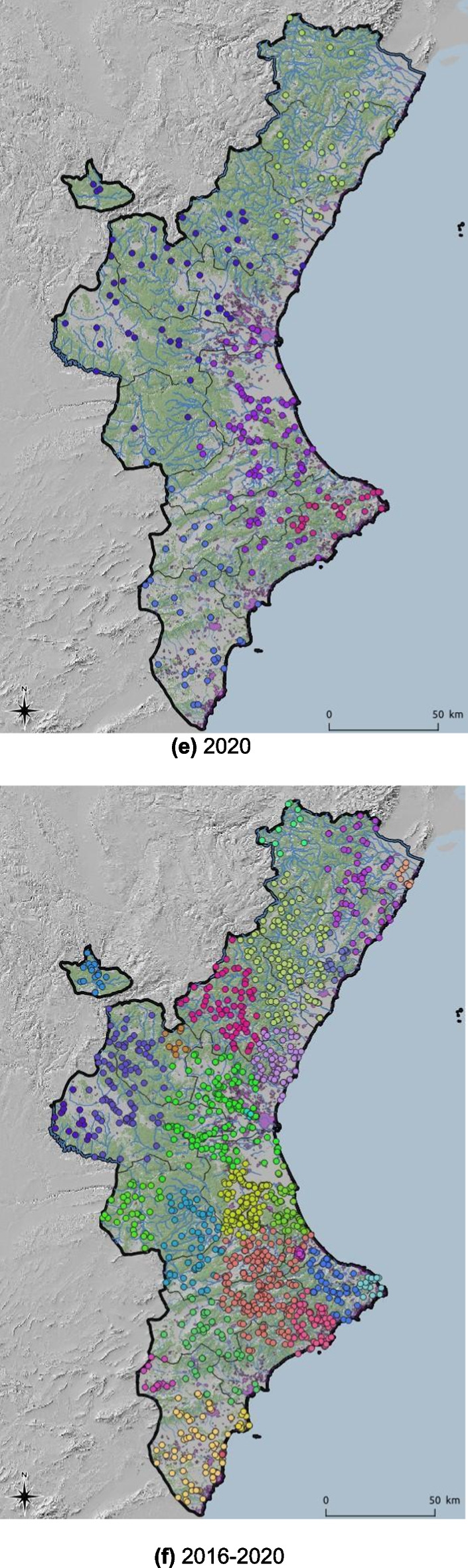
Distribution of wildfires from 2016 to 2020 and 2016–2020 according to cluster analysis (each color represents unique clusters)

**Fig. 12:**
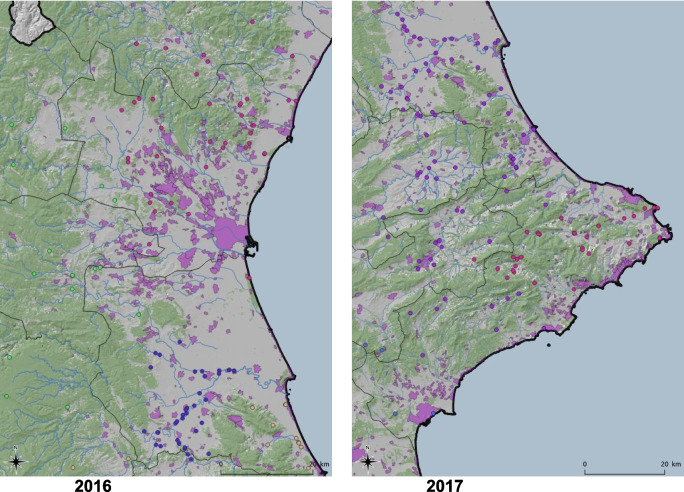
Distribution of clusters in zoomed zone for 2016 and 2017 (each color represents unique clusters)

As already depicted in the graphs in Fig. 2, the highest density is observed in the southeastern part of the study area. The last figure represents the spatial clusters for all five study years together. As mentioned in the Introduction section and represented in Fig. 11, fires are located from south to north throughout the study area. The maps in Fig. 11 show the same distribution as Fig. 2, but now consider the cluster analysis. Therefore, each color represents the fires that belong to the same cluster. In addition, to better understand these maps concerning the type of land use, forestry areas are represented in green, rivers are identified with blue lines, and urban areas are shown in purple (Fig. 12).

**Fig. 13:**
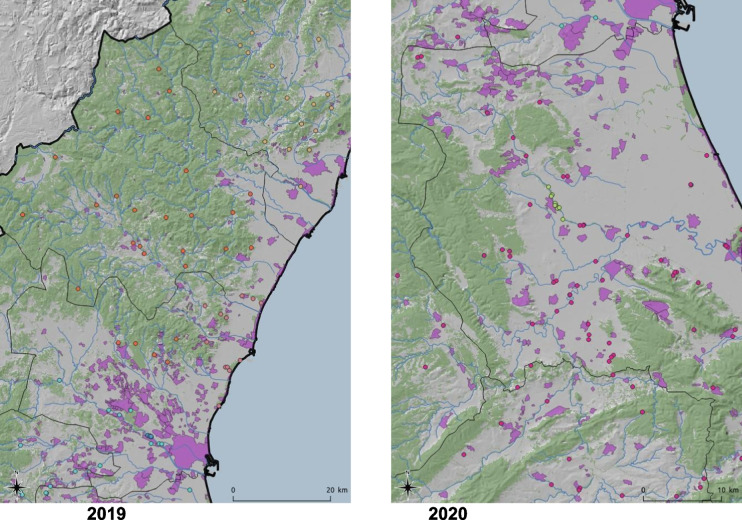
Distribution of clusters in zoomed zone for 2019 and 2020 (each color represents unique clusters)

Regarding the distribution of wildfires in clusters, it is observed that, except for 2016, wildfires are clearly distributed in groups throughout the entire territory, especially in 2018 and 2019. For 2016, wildfires do not seem to share the same pattern, as no well-defined group distribution appears throughout the study area. The southern forested area, therefore, has a very well-represented group. Moving to the central part of the study area, from 2017 to 2019 wildfires went from east to west. By looking at the center of the map in the province of Valencia, reveals the following locations of clusters: one in the southeast, another in the northeast, and finally another in the western area (inland). In the north, basically two clusters (2018–2019) divide this area into two parts, one further north and one further south of this northern part of the study area. In contrast, for this same area (the north), in 2017, the wildfires divided this area between coastline and inland. Records for 2020 show similar wildfire patterns to those of 2019, but with fewer wildfires in each cluster (Fig. 13). This clustering division changes from year to year, and therefore, a further analysis should be performed to have a relation between spatial distribution, weather and causes.

Table 1 summarizes covariate distributions, showing wide variation in burned area, with many small fires and few large ones. Precipitation (Fig. 7) and temperature (Fig. 8) varied across clusters (Fasullo et al., 2018). Supplementary Figures S1 and S2 show precipitation and temperature trends for 2016 and 2020.

Land use analysis (Tables 4, 5, 6, 7 and 8) revealed that wildfires primarily burned scrub/herbaceous vegetation, permanent crops, and forests. In 2019, permanent crops slightly outnumbered scrub/herbaceous vegetation. Heterogeneous agricultural areas accounted for ~ 13% of burned land. Clusters differed between natural and agricultural land uses, with some clusters (e.g., 2017 Clusters 8 and 12) burning no forest land.

**Table 4:** CORINE Land Cover wildfire distribution by cluster in 2016

	Clusters															
Type of land cover	1	2	3	4	5	6	7	8	9	10	11	12	13	14	Grand total	Percentage
Rice fields	1			1											2	1
Mixed forest							1	1							2	1
Coniferous forest	11	1	4	3	1		5	3		3	2	9		12	54	16
Broad-leaved forest						2	3			1				1	7	2
Sparsely vegetated areas	1	1													2	1
Fruit trees and berry plantations	8	5	2	42		1	1			2	3	3		1	68	20
Inland marshes				1											1	0
Marshlands		1									3				4	1
Transitional woodland-shrub	1		3		2	1	2								9	3
Complex cultivation patterns	1		4			4	7	1		2		2		3	24	7
Olive groves							2			6		1			9	3
Natural grasslands	4	1	8	6	2	2	10	1		3	1	5	2	5	50	15
Beaches, dunes, sand				1											1	0
Grasslands			1	1			2								4	1
Salt marshes			1												1	0
Continuous urban fabric			1												1	0
Discontinuous urban fabric	2		1		2		1	3						1	10	3
Land mainly devote to agriculture, with significant areas of natural vegetation	2	2	4	2	1	2	3	1	2			2			21	6
Permanently irrigated land			1			1					1		2		5	1
Non-irrigated arable land					2										2	1
Sclerophyllous vegetation	5	1		4	3	7	5	5	5	7	2	9		6	59	17
Vineyards					3		2								5	1
Total fires	36	12	30	61	16	20	44	15	7	24	12	31	4	29	341	100

**Table 5:** CORINE Land Cover wildfire distribution by cluster in 2017

	Clusters							
Type of land cover	1	2	3	4	5	6	Grand total	Percentage
Rice fields				5			5	1
Coniferous forest	18	15	26	19		1	79	23
Broad-leaved forest		4					4	1
Sparsely vegetated areas		1		2			3	1
Fruit trees and berry plantations		7	4	44	1	10	66	19
Marshlands				1			1	0
Transitional woodland-shrub	4	2	3	4		2	15	4
Complex cultivation patterns		3	3	4		4	14	4
Olive groves		1	1	5		1	8	2
Natural grasslands	9	4	8	18	6	6	51	15
Beaches, dunes, sand		1			3	1	5	1
Pastures				1		2	3	1
Salt marshes	1						1	0
Continuous urban fabric	3						3	1
Discontinuous urban fabric		2	1	1			4	1
Land mainly devoted to agriculture, with significant areas of natural vegetation	1	1	6	14		9	31	9
Permanently irrigated land	2						2	1
Non-irrigated arable land		1					1	0
Sclerophyllous vegetation	1	15	6	18		5	45	13
Vineyards			2				2	1
Industrial or commercial units				1		1	2	1
Urban green zones	1						1	0
Total fires	40	57	60	137	10	42	346	100

**Table 6:** CORINE Land Cover wildfire distribution by cluster in 2018

	Clusters									
Type of land cover	1	2	3	4	5	6	7	8	Grand total	Percentage
Rice fields				1					1	0
Mixed forest						1	1		2	1
Coniferous forest	16	8	6	12	12	1	14	8	77	21
Broad-leaved forest	2			1					3	1
Sparsely vegetated areas	1								1	0
Fruit trees and berry plantations	3	3		32	8	2	1	10	59	16
Inland marshes					1				1	0
Marshlands		3				1			4	1
Transitional woodland-shrub	5	1	2	2	3	1	2	1	17	5
Complex cultivation patterns	3	1		3	4	3		7	21	6
Olive groves	3			5		2			10	3
Natural grasslands	16	4	3	11	10	5	3	2	54	14
Beaches, dunes, sand				1		1			2	1
Pastures		2			1			3	6	2
Bare rocks					1				1	0
Continuous urban fabric								1	1	0
Discontinuous urban fabric		1		1	2	2	1	2	9	2
Land mainly devoted to agriculture, with significant areas of natural vegetation	8	2	1	8	8	2	1	4	34	9
Permanently irrigated land	1	1					4		6	2
Non-irrigated arable land				1	1				2	1
Sclerophyllous vegetation	12	2	1	13	6	6	3	9	52	14
Vineyards							10		10	3
Industrial or commercial units		1							1	0
Burned areas				1					1	0
Total fires	70	29	13	92	57	27	40	47	375	100

**Table 7:** CORINE Land Cover wildfire distribution by cluster in 2019

	Clusters							
Type of land cover	1	2	3	4	5	6	Grand total	Percentage
Rice fields	1						1	0
Coniferous forest	22	1	11	5	4	6	49	18
Broad-leaved forest			2		1	2	5	2
Sparsely vegetated areas	1						1	0
Fruit trees and berry plantations	20	30	7	1	3	4	65	24
Inland marshes	1						1	0
Coastal lagoons	2						2	1
Transitional woodland-shrub	3		1	3		4	11	4
Complex cultivation patterns	5		4	1	2	6	18	7
Olive groves	6				1		7	3
Natural grasslands	5	1	10	1	8	5	30	11
Beaches, dunes, sand			1		1		2	1
Pastures	1		1	1	1	1	5	2
Bare rocks	2						2	1
Continuous urban fabric	1	2		3			6	2
Discontinuous urban fabric	2		1				3	1
Land mainly devoted to agriculture, with significant areas of natural vegetation	9	1	5	1		1	17	6
Permanently irrigated land			2	1		1	4	1
Non-irrigated arable land	1					1	2	1
Sclerophyllous vegetation	8	2	8	1	9	5	33	12
Vineyards				1		7	8	3
Total fires	90	37	53	19	30	43	272	100

**Table 8:** Types of land use in CORINE Land Cover

Code CLC 09	Land use
10	Urban and suburban areas
20	Temporary rainfed crops
30	Temporary irrigated crops
40	Forest
50	Heathlands and sclerophyll vegetation
60	Prairies
70	Areas of sparse vegetation
80	Areas of low vegetation and soil regularly flooded
90	Bare areas
100	Complex crops
110	Agro-forestry systems
120	Mosaic of natural vegetation (herbaceous, bushes and trees)
200	Water bodies

## Discussion

Cluster analysis confirmed that wildfires with shared characteristics group together, providing robust insights into spatial and environmental patterns. The methodology validated intuitive observations and highlighted the heterogeneity of wildfires over time. Clusters were strongly influenced by orographic features, such as mountains and river basins, dividing the region into flat and mountainous areas (Júnior et al., 2022; Sarala et al., 2022; Saxe et al., 2021).

The distribution of wildfires varied annually, with 2016 showing less defined clustering compared to 2018 and 2019. Southern forested areas exhibited clear clustering, while central areas showed east-to-west wildfire spread. Northern areas were divided into coastal and inland clusters in 2017 and further subdivided in 2018–2019. The 2020 patterns resembled 2019 but with fewer fires.

Land use played a significant role in wildfire distribution, with scrub/herbaceous vegetation and permanent crops being the most frequently burned. Clusters near rivers, particularly in areas with Arundo donax L., highlighted the continued use of fire for land management despite its illegal status and associated risks (Jiménez-Ruiz et al., 2021).

The study underscores the importance of considering environmental covariates (e.g., elevation, slope, aspect, precipitation, temperature) and land use in wildfire analysis. The findings provide a foundation for targeted wildfire management strategies, particularly in high-risk areas identified through cluster analysis. The BIC values and model selections (Zhang & Di, 2020) further support the robustness of the clustering methodology.

**Fig. 14:**
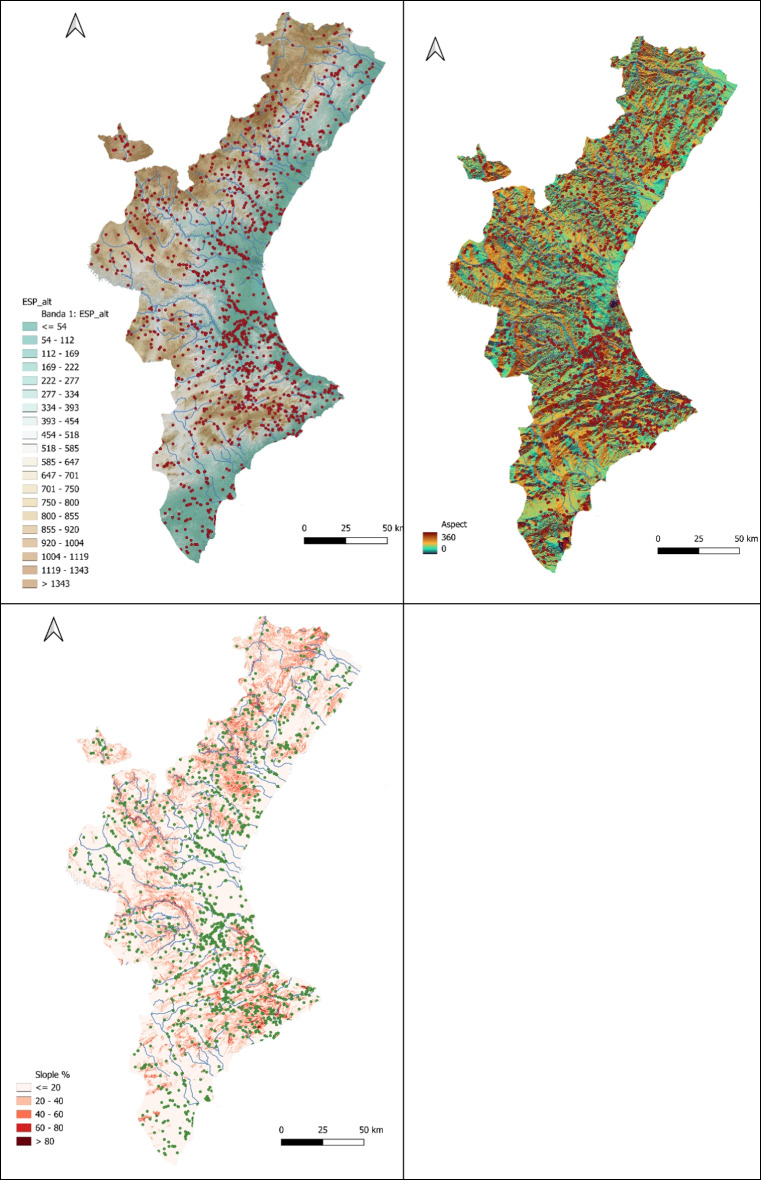
Altitude, aspect, and slope figures

Human management of land cover and the traditional use of fire are closely related (Badia et al., 2019). In this study, we explore the identification of land cover across wildfire clusters. While factors such as aspect, altitude, and slope can help explain wildfire behavior, land cover—particularly human-influenced land cover—offers valuable insights into the social dynamics surrounding wildfire events (Fig. 14).

The use of GMM in this study was driven by their flexibility and ability to model complex, multi-modal data distributions. GMMs allow for the estimation of clusters based on probabilistic distributions, making them particularly well-suited for identifying hidden structures within the wildfire data. The application of the mclust package for clustering was chosen due to its robust set of tools for model-based clustering, density estimation, and discriminant analysis, which align with the study’s objective of understanding the spatial distribution and characteristics of wildfires over time. Moreover, GMMs were validated through the BIC, which provides a statistical measure to assess the trade-off between model fit and complexity. By selecting the model that minimizes the BIC, we ensure that the clustering solution reflects both the underlying data distribution and avoids overfitting. The iterative process of selecting the optimal number of clusters and the appropriate model (e.g., VII, VEI) across multiple years highlights the dynamic nature of the data and ensures that the clustering methodology remains adaptable to the changing characteristics of wildfires.

A limitation of the current study is the exclusive focus on GMMs for clustering. In future research, it would be valuable to expand upon the findings by conducting a comparative analysis with other clustering techniques, such as *k*-means or hierarchical clustering, to better understand their relative strengths and limitations in the context of wildfire prediction. This study focused on exploring the applicability of GMMs as a robust and well-established method for identifying spatial and temporal wildfire patterns. The use of GMMs, supported by the mclust package and BIC, serves as an initial step in demonstrating the feasibility of this approach. A more comprehensive comparison with alternative methods could provide additional insights and refine the clustering process in future work, building on the foundation established here.

## Conclusions

This work shows the results obtained thanks to the application of statistical techniques that have been little used up until now in the literature in the field of wildfires. The package of functions used, the *mclust* library implemented in R, has made it possible to characterize and analyze the spatial distribution of wildfires, creating clusters that share common characteristics and, therefore, can help better understand their wildfires. Thus, in this paper, the finite GMM methodology has been presented to analyze the behavior of fires in terms of clustering in order to know how fires are grouped in the Valencian Community according to their spatial arrangement. The results show that fire occurrences in the study area are closely linked to the same set of covariates along years but that the way those covariates influence fire occurrences varies along years. The most relevant covariates associated to clustering of fire occurrences were topography and land cover. Temperature and precipitation did not play a significant role in clustering, probably because most of the fires occur during hot summer months. Cluster analysis proved to be useful to understand the main particularities of fire occurrences in areas with similarities in terms of land cover, providing key information for improvements of wildfire management policies. Our results are shown graphically in two maps of spatial coordinates to give a clear and comprehensive message to fire managers. In the specific case of the Valencian Community, located in the Mediterranean area of Spain, a clear clustering pattern is observed and helps to understand which areas are potentially prone to occurrences of scrub fires, wildfires, or fires related to agriculture.

The current study shows a clear pattern of wildfire clusters near the paths followed by rivers. This can be an interesting domain for exploration by future spatial researchers. It is worth noting that fewer wildfire clusters were reported in 2020 because of the pandemic lockdown.

The methodology can be used in future studies to understand which variables favor ignition in specific areas and can also shed light on a smaller scale, that is, to work on elements of the fires in municipalities or districts where fire outbreaks occur. The study helps to depict how wildfires are grouped, whether due to negligence or arson. All this will be addressed in future studies in which other variables of interest are available that help understand the behavior of these environmental phenomena. Moreover, if the analysis includes the cause of the wildfire, it can help detect regions in which an arsonist may act. In this way, it can become possible to detect areas with similar characteristics of forest ignition, allowing the implementation of improvements in wildfire prevention strategies, and provides a baseline to help researchers understand wildfire behavior with emphasis on the type of land cover and other covariates using cluster analysis techniques.

Another aspect to consider is that, while this study primarily applied GMM as a robust methodology for wildfire analysis, future research will explore comparisons between GMMs and other clustering techniques to further refine and validate the findings.

## Supplementary Information

Below is the link to the electronic supplementary material.

Supplementary file1 (DOCX 96 KB)

## Data Availability

The data that support this study are available in Estadística i investigació d'incendis forestals at https://agroambient.gva.es/va/web/prevencion-de-incendios/estadistica-de-incendios-forestales/ 25.02.2022.
