# Supplementary material for: Classification of wildfires in relation to land cover types and associated variables by applying cluster analysis: a case study in the Iberian Peninsula
Source: Environ Monit Assess. 2025 May 3;197(6):619. doi: 10.1007/s10661-025-14053-y (PMC12049408; doi:10.1007/s10661-025-14053-y)
Supplement: Supplementary file 1 — Supplementary file1 (DOCX 96 KB) [file 10661_2025_14053_MOESM1_ESM.docx]

**Supplementary Materials**

**Boxplot for Annual Precipitation**


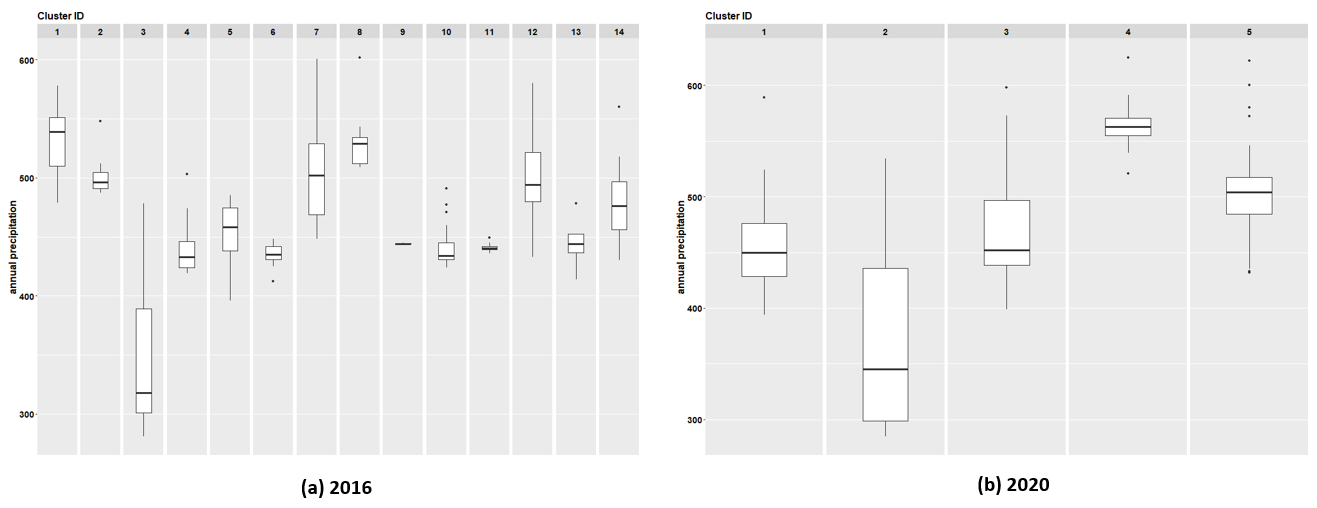


**Figure S1.** Boxplot of annual precipitation per individual cluster

As a showcase, we report the initial year (2016) and the final year (2020)

of the entire study period, on the left and on the right, respectively.

**Boxplot for Mean Annual Temperature**


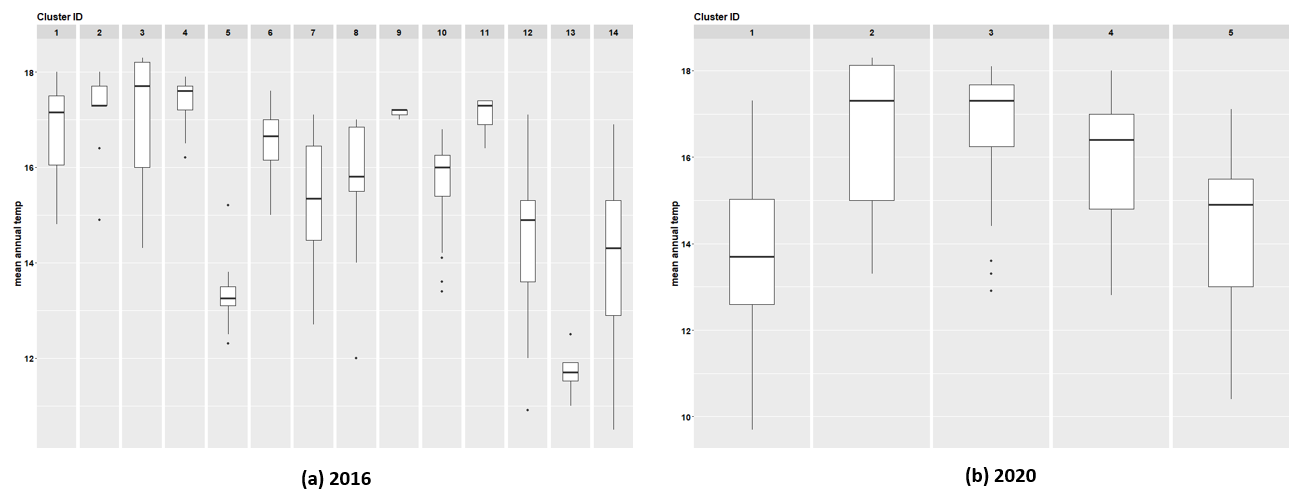


**Figure S2.** Boxplot of annual mean temperature per individual cluster

As a showcase, we report the initial year (2016) and the final year (2020)

of the entire study period, on the left and on the right, respectively.
